# Exosomes in Head and Neck Squamous Cell Carcinoma

**DOI:** 10.3389/fonc.2019.00894

**Published:** 2019-09-18

**Authors:** Cheng Xiao, Fang Song, Yu Long Zheng, Jiong Lv, Qiang Feng Wang, Nong Xu

**Affiliations:** ^1^Department of Medical Oncology, College of Medicine, The First Affiliated Hospital, Zhejiang University, Hangzhou, China; ^2^Department of Anesthesiology, College of Medicine, The First Affiliated Hospital, Zhejiang University, Hangzhou, China; ^3^Department of Oral and Maxillofacial Surgery, College of Medicine, The First Affiliated Hospital, Zhejiang University, Hangzhou, China

**Keywords:** exosomes, head and neck squamous cell carcinoma, tumor microenvironment, biomarkers, therapy resistance

## Abstract

Exosomes are small membranous vesicles that contain proteins, lipids, genetic material, and metabolites with abundant information from parental cells. Exosomes carry and deliver bioactive contents that can reprogram the functions of recipient cells and modulate the tumor microenvironment to induce pathological events through cell-to-cell communication and signal transduction. Tumor-derived exosomes (TDEs) in head and neck squamous cell carcinoma (HNSCC) are involved in most aspects of cancer initiation, invasion, progression, immunoregulation, therapeutic applications, and treatment resistance. In addition, HNSCC-derived exosomes can be used to obtain information on diagnostic and therapeutic biomarkers in circulating blood and saliva. Currently, the biology, mechanisms, and applications of TDEs in HNSCC are still unclear, and further research is required. In this review, we discuss various aspects of exosome biology, including exosomal components, exosomal biomarkers, and molecular mechanisms involved in immunoregulation, cancer metastasis, and therapy resistance. We also describe recent applications to update our understanding of exosomes in HNSCC.

## Introduction

Head and neck cancer (HNC) is one of the most widespread malignancies worldwide. Although continual progress has been made in the treatment of HNC, the 5-year overall survival rate of advanced HNC remains low at approximately 50% (1, 2). HNC frequently develops from mucosal surfaces of the mouth, including the oral cavity (tongue, lip, buccal, gingiva, and palate), oropharynx, larynx, and perioral skin carcinoma (3). More than 90% of HNCs are head and neck squamous cell carcinoma (HNSCC). The exact etiology of HNSCC remains unclear; however, tobacco and alcohol consumption are major risk factors for HNSCC, as demonstrated in epidemiological studies. Mucosal human papilloma virus (HPV) is also related to a subset of HNSCCs; ~25.9% of HNSCCs are HPV positive, whereas the prevalence of HPV in oropharyngeal squamous cell carcinoma (SCC) is 34.1%, which is higher than that in oral SCC (4).

Exosomes, which were first discovered in 1983, are 30–150 nm mature double membrane multivesicular bodies (MVBs) originating from the endosomal pathway (5). Exosomes exist in the extracellular space and in liquids, such as blood, urine, and saliva (6). Exosomes are associated with many physiologic aspects of the disease via intercellular communication and signal transduction, indicating that exosomes have potential clinical applications as biomarkers and therapeutic targets. Tumor-derived exosomes (TDEs) contain a cytomembrane, proteins, nucleic acids, lipids, and other substances from parental tumor cells (7). Moreover, exosomes are known to be involved in nearly all stages of cancer (8–11). Growing evidence has demonstrated that TDEs participate in the development, progression, and treatment of cancer by mediating intercellular communication and signal transduction (12, 13). The bioactive components of HNSCC-derived exosomes, such as microRNAs, transcription factors, and oncogenic proteins, play key roles in mediating tumorigenesis, tumor microenvironment reprogramming, immune tolerance, promoting metastasis, and therapy resistance. For example, intracellular annexin 1 (ANXA1) regulates epidermal growth factor receptor (EGFR) activity and alter the release of EGFR-containing TDEs in HNCs (14). Exosomes produced by hypoxic oral SCC cells deliver viral *miR-21* to normoxic cells, inducing the epithelial-mesenchymal transition (EMT) to promote cell migration and invasion (15). Another study showed that exosomal nuclear factor-κB-activating kinase-associated protein 1 (NAP1) derived from oral cancer promotes the cytotoxicity of natural killer (NK) cells via activation of the interferon regulatory factor (IRF-3) signaling pathway in recipient cells (16). In addition, *miR-34a-5p* in cancer-associated fibroblast (CAF)-derived exosomes in oral SCC stimulates the proliferation and metastasis of oral cancer cells through the AKT/glycogen synthease kinase-3β/β-catenin/Snail signaling cascade (17). A recent study demonstrated that thrombospondin 1 derived from oral SCC exosomes is also involved in the polarization of macrophages to M1-like tumor-associated macrophages and promotes the invasion of cancer cells (18). HNSCC-derived exosomes containing EphrinB1 may manipulate the tumor microenvironment through induction of tumor innervation (19). Additionally, Sento demonstrated that oral SCC-derived exosomes promote tumor growth by activating the phosphatidylinositol 3-kiase (PI3K)/AKT, mitogen-activated protein kinase (MAPK)/extracellular signal-regulated kinase (ERK), and c-Jun N-terminal kinase-1/signal transducer and activator of transcription (STAT) 2 pathways (20). Emerging evidence has supported the vital role of TDEs in the development, progression, and treatment of HNSCC.

In this review, we summarize many aspects of exosome biology and functions in HNSCC.

## Biogenesis, Features, and Components of Exosomes

### Inward Budding and MVB Formation

Different types of vesicles, including extracellular vesicles (EVs), MVBs, and exosomes, have been described and often labeled interchangeably in many previous studies. Although these different types of vesicles share overlapping features, they have distinct morphologies, properties, biogenesis mechanisms, and functional roles. Plasma membrane components and enclosing cytosolic components are incorporated into the invaginating membrane, resulting in the formation of early endosomes (21). Exosomes typically originate from inward budding from the membrane and are then released into the extracellular space via activation of Ca^2+^-dependent or Rab-GTPases (22). Briefly, exosomes are generated from early endosomes, mature into MVBs, and are then secreted into the extracellular space upon fusion with the plasma membrane. First, exosomes start as early endosomes, which are formed by endocytosis of the plasma membrane. The biogenesis of exosomes and sorting of functional cargo is precisely regulated by certain mechanisms involving multiple factors. The most commonly described pathway for exosomes biogenesis is the endosomal sorting complex required for transport (ESCRT) machinery. Four types of ESCRTs (ESCRT-0–III) are involved in regulating MVB formation, vesicle budding, and protein cargo sorting (23). The ESCRT mechanism is initiated and sequestrated by ubiquitinated proteins to domains of the endosomal membrane via ubiquitin binding subunits of ESCRT-0 in the endosomal membrane, then interacting with the ESCRT-I and ESCRT-II complexes inducing membrane deformation into buds. Finally, the ESCRT-III complex separates from the MVBs membrane (23–25). However, the machinery that drives the load of protein cargo into ESCRT-dependent exosomes is still unclear.

Cells also utilize ESCRT-independent pathways, involving insphingosine-1-phosphate, ceramide, tetraspanin-enriched microdomains, and sphingomyelinase, for exosome production and release (26–28). These ESCRT-independent mechanisms may participate in promoting domain-induced budding, sorting of bioactive molecules into exosomes, segregation of cargo within the endosomal membrane, and exosome formation.

The ESCRT-dependent and -independent mechanisms of exosome release are based on the cell origin. In addition, membrane proteins of lysosomes and late endosomes may be important for the biogenesis and secretion of exosomes (29).

### Regulated Secretion and Intercellular Interactions

Exosome secretion is involved in various signaling pathways. For example, the key regulatory role of RAB family proteins in trafficking intracellular exosomes was demonstrated by Colombo et al. (30). Another report showed that the Wnt pathway is particularly important for the dysregulation of exosome release in cancer cells (31). Additionally, the secretion of exosomes is mediated through exocytosis-associated molecular motors and cytoskeletal proteins (32). Spontaneous secretion of exosomes usually occurs at the steady state; however, some conditions are known to stimulate exosomes. Indeed, cell intrinsic signals are known to enhance the release of high levels of TDEs from cancer cells via activation of oncogenic signaling pathways or regulation of membrane fusion machinery (33). In addition, evidence suggests that microenvironmental conditions enhance exosome release from cancer cells (15, 34). Exosomes are then released into the extracellular environment through exocytosis or degraded by fusing with lysosomes. As previously described, Rab GTPases are essential regulators of exosome secretion. Furthermore, several studies have shown that soluble N-ethylmaleimide-sensitive component attachment protein receptor complexes, which are involved in membrane fusion machinery, may affect the secretion of exosomes (35–37).

**Figure 1:**
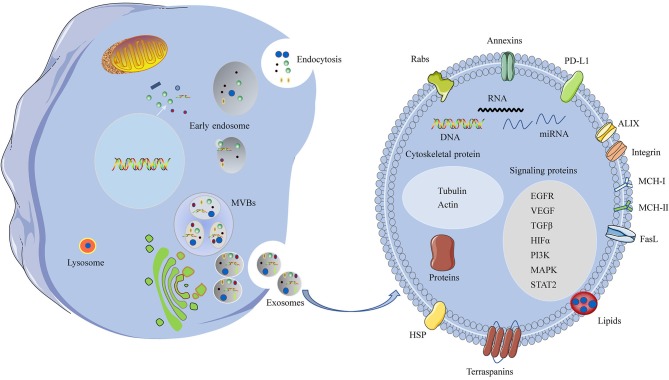
A schematic representation of biogenesis and components of the HNSCC-derived exosomes. Invagination of the plasma membrane form early endosomes, followed by budding of payload into the endosomal membrane to form multivesicular endosomes (MVBs). Maturation of the late endosome through fusion with the plasma membrane and release of exosomes. Some of the late endosome fuse with lysosomes for lysosomal degradation. The HNSCC-derived exosomes contain various cellular ingredients, such as nucleic acids, lipid, and proteins, which act as key molecules of signaling transduction.

Ultimately, exosomes are internalized by recipient cells through receptor-mediated endocytosis, pinocytosis, phagocytosis, or fusion with the cell membrane, resulting in delivery of molecular and genetic components into the recipient cells (30). Exosomes target recipient cells after secretion into the extracellular space and then induce changes in downstream signaling pathways. The specificity of target recipient cells is dependent on the type of ligand/receptor pairs present on exosomes and recipient cells; and a study provides further insight that glycans are key players in the process of exosomes uptake (38). A schematic representation of exosome biogenesis and secretion pathways is shown in Figure 1.

## Features and Components of Exosomes

Typical exosomes exhibit a particular biconcave or cup-like shape and are observed as spheroids under transmission electron microscopy (39). Exosome contents are rich in nucleic acids, cytosolic/endosome proteins, and cytoskeleton components, which have unique biological activities (40). The components of TDEs are distinct from those of exosomes released from healthy cells. TDEs deliver functional cargo, including oncogenes and oncogenic proteins that can exert biological activities. The functional cargoes, such as protein cargo, RNA cargo, and DNA cargo, of TDEs in the tumor microenvironment have an important role in promoting cancer progression and metastasis (Figure 1) (33). Protein cargoes, including different oncoproteins, immunomodulatory molecules, and growth factors, act as mediators of tumorigenesis. TDEs also contain abundant nucleic acids, such as microRNAs (miRNAs), mRNA, long noncoding RNAs, and DNAs, which deliver genetic information (41–43).

Many researchers have analyzed the functions of miRNAs in exosomes, and various specific miRNA cargoes and extracellular components have been detected in exosomes. These observations highlight the specificity of exosomal miRNAs, distinct miRNA signatures of exosomes, and various functions of exosomal miRNAs. A series of experiments have analyzed the function of the miRNAs in exosomes since their initial description (44). It has been reported that there was a selection of specific miRNA cargoes and extracellular exports in exosomes (30). These observations highlight the specificity of exosomal miRNAs, distinct miRNA signatures of exosomes, and various functions of exosomal miRNAs. Exosomal miRNAs are associated with a variety of pathological activities, including tumorigenesis, invasion, progression, angiogenesis, metastasis, and chemoresistance. However, not all exosomal miRNAs are implicated in tumor-supportive mechanisms. miRNAs of exosomes are highly functional, with roles in intercellular communication and tumor microenvironment regulation, indicating that exosomal miRNAs play important roles in diagnostic and therapeutic applications.

In addition, exosomes contain ceramides, lipids, sphingolipids, cholesterol, and glycerophospholipids. Because exosomes originate from the fusion of endocytic compartments with the plasma membrane, the protein, lipid, and double lipid layer compositions can be used to identify exosomes. The most commonly identified markers are ALIX and tetraspanins, such as CD9, CD63, CD81, and CD82 (23).

## Functions of Exosomes in HNSCC

Exosomes have been identified as bioactive and informative nano-sized MVBs that influence many aspects of the development and progression of HNSCC. Here, we discuss recent findings of the mechanisms through which HNSCC-derived exosomes modulate the immune response, tumor microenvironment, cell-to-cell communication, and tumor invasion. We also describe exosomes as potential biomarkers and discuss their applications in cancer therapy and therapy resistance.

## Exosomes as Potential Biomarkers

In the clinical setting, some relevant biomarkers have been shown to influence treatments for patients with HNSCC. Several reports have shown that liquid biopsies, including biopsies of circulating tumor DNA, circulating tumor cells, and exosomal miRNAs, can have potential clinical applications in HNC (45). Moreover, HPV status is a prognostic factor in HNSCC; patients with HPV positivity have better responsiveness to chemotherapy and radiotherapy and are more susceptible to immune surveillance. In addition, miRNAs are independent prognostic markers for patients with HPV-negative HNSCC (46), and EGFR overexpression is associated with poorer prognosis and outcomes in HNSCC (47). However, further studies are needed to establish biomarkers for staging HNSCC and facilitating therapeutic decision-making.

Exosomes modulate various pathological activities to promote cancer cell growth, invasion, and distant metastasis. TDEs also carry valuable genomic and proteomic information and can provide information regarding alterations in genomic and proteomic profiles of exosomes from patients with cancer in response to anticancer therapies (48). Such proteomic and genetic components are well-protected within the lipid bilayer and can be preserved without significant loss of functional profiling. Therefore, exosomes may serve as promising markers for monitoring cancer progression and therapeutic responses (49).

TDEs can be obtained from blood or saliva of patients with HNSCC, indicating that TDEs may represent a real-time, non-invasive, clinically relevant biomarker of cancer progression, and treatment responses (45, 50–53). For example, the number of exosomes in the plasma has been shown to be a prognosis indicator for HNSCC. Gimzewski reported that elevated exosome numbers, exosome sizes, and interexosomes were detected in the saliva of patients with oral cancer (54). In addition, patients with HNSCC with advanced-stage disease and shorter overall survival usually exhibit elevated levels of exosomes in the plasma, indicating that plasma exosomes in HNSCC may have applications in monitoring tumor progression (52, 54). One previous study demonstrated that TDE signatures could serve as candidate biomarkers for early cancer diagnosis, monitoring, and surveillance in HPV-16-associated oropharyngeal (55). Moreover, a non-invasive method involving Fourier-transform infrared spectroscopy of salivary exosomes was shown to have high sensitivity and specificity in the diagnosis of oral cancer (56).

Non-coding RNAs from plasma exosomes have been extensively studied as potential biomarkers in HNSCC because they are derived from whole tumor cells and may therefore represent whole cellular RNAs (57, 58). Several studies have reported that cancer cells can selectively pack selected miRNAs into exosomes, and these selective exosomal miRNAs then act as tumor suppressors or oncogenes in HNSCC (59–61). Additionally, a hypoxic microenvironment can stimulate oral SCC to generate *miR-21*-rich exosomes, which are associated with lymph node metastasis (15). Serum exosomal *miR-21* and homeobox transcript antisense RNA (*HOTAIR*) are also significantly associated with the clinical characteristics of laryngeal SCC (59). In a previous study, elevated CAF-derived exosomal *miR-196a* levels were shown to be correlated with cisplatin resistance in HNSCC through targeting cyclin-dependent kinase (CDK) N1B and inhibitor of growth family member 5 (ING5), indicating that this miRNA may serve as a promising predictor of cisplatin resistance and poor survival in HNSCC (60). A study by Zhou and colleagues revealed that there were significant differences in expression between exosomal miRNAs and cellular miRNAs in laryngeal SCC (61). Furthermore, oral cancer-derived salivary exosomal *miR-512-3p* and *miR-412-3p* may serve as potential biomarkers (62). In another example, Inazawa and colleagues found that exosomal *miR-1246* induces cell motility and invasion through directly targeting differentially expressed in normal vs. neoplastic/MAPK-activating death domain-containing 2D in oral SCC (63). Collectively, these results suggested that exosomal miRNAs could serve as excellent diagnostic and prognostic biomarkers.

Analysis of exosomal proteins is a novel tool for developing exosomes as potential biomarkers for HNSCC. More than 80% of HNSCCs exhibit overexpression of EGFR in the membrane, and hyperactivity EGFR plays an important role in tumorigenesis development and drug-resistance mechanisms by activating various signaling pathways, including the PI3K/AKT, RAS/MEK/ERK, and Janus kinase (JAK)/STAT pathways, in HNSCC. A recent study showed that EGFR can be secreted from cells via the transport of exosomes and that these EGFR-containing exosomes have the ability to regulate autocrine VEGF production in endothelial cells (64). Exosomal EGFR mediates metastasis and tumor immunity in lung cancer (65). ANXA1, a tumor suppressor in HNSCC, regulates EGFR activity and exosomal phospho-EGFR release, revealing that exosomal EGFR and phospho-EGFR may be prognostic biomarkers in HNSCC (14). Additionally, the levels of exosomal EGFR and phospho-EGFR are reduced after cetuximab treatment, indicating that exosomes can serve as biomarkers to monitor cetuximab treatment (66).

Analysis of exosome protein profiles showed that the characteristics and functions of exosomes from HPV-positive/-negative HNC differed significantly. HPV-positive exosomes had low p53 levels and did not contain cyclin D1, but did harbor p16, E6/E7, and the T-cell inhibitory protein PTPN11 (67). A recent study revealed that proteome analysis of salivary extracellular vesicles may yield prognostic biomarkers for oral SCC (68).

The microenvironment of HNSCC is highly immunosuppressive, and the programmed cell death (PD)-1/PD-ligand 1 (PD-L1) pathway plays an important role in HNSCC. High levels of PD-L1 are associated with poor outcomes in various types of cancer, including HNSCC. PD-1 checkpoint inhibitors were found to be safe and effective in platinum-refractory recurrent or metastatic HNSCC (69, 70). Furthermore, a study by Whiteside and colleagues indicated that PD-L1^+^ exosomes in the plasma were related to immune suppression and disease progression. Additionally, blocking PD-L1^+^ exosome signaling to PD-1^+^ T cells attenuated immune suppression in patients with HNSCC (71). In another study, Ferris reported that JAK2/STAT1 signaling was involved in EGFR-mediated immune evasion in HNSCC and that therapies targeting this signaling pathway may be beneficial for blocking PD-L1 upregulation in HNSCC (72). Moreover, elevated heat-shock protein 90 levels in TDEs have been reported to serve as potential biomarkers for clinical stage and prognosis in patients with oral cancer (73). A recent study showed that plasma-derived exosomes were associated with disease progression of HNSCC after separation into CD3^+^ and CD3^−^ fractions (74). Furthermore, HNSCC-derived exosomes have been shown to exhibit synergistic interactions with invadopodia, indicating that exosomes play key roles in promoting cancer invasion (35). TDEs inducing transcriptome reprogramming can cause cancer-associated pathologies in HNSCC, including angiogenesis, immunoregulation, and metastasis; these functional differences in HNSCC may serve as candidate markers (75).

**Figure 2:**
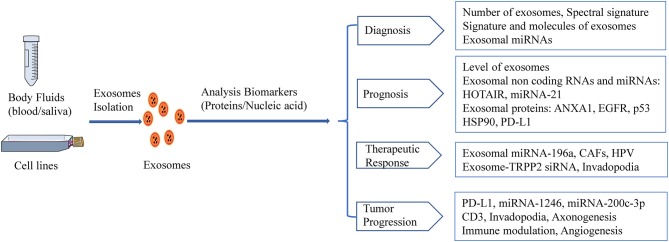
HNSCC-derived exosomes act as biomarkers. Exosomal biomarkers screening of diagnostics, prognosis, or therapeutics in HNSCC.

A growing body of evidence has shown that the characteristics of TDEs and exosomal components (e.g., exosomal miRNAs, exosomal proteins) may serve as potential noninvasive biomarkers for the detection, monitoring, and treatment of HNSCC (Figure 2). However, methods for TDE isolation and separation are complicated and time-consuming, and additional studies of TDEs as non-invasive biomarkers are needed.

## Roles in Regulating the Tumor Microenvironment

### Immune Cells

The tumor microenvironment is formed by stromal cells and is associated with malignant progression (76). Moreover, TDEs play important roles in regulating the tumor microenvironment of HNC (77). The immunological activities of exosomes are related to many aspects of immune regulation, including antigen presentation, immune activation, immune surveillance, and immune suppression. Tumor-infiltrating myeloid-derived suppressor cells (MDSCs), tumor-associated macrophages (TAMs), and regulatory T cells (Tregs) are known mediators of the immunosuppressive microenvironment and limit the efficacy of immune therapy in HNC (78). Exosomes that contain immunosuppressive molecules can facilitate immunosuppression in cancer, which helps cancer cells escape from immune responses, thereby promoting tumorigenesis (79, 80). Additionally, exosome-associated bioactive proteins and RNAs have been shown to regulate the immune system (81, 82). Exosomes may mediate immune suppression through directly/indirectly inhibiting the functions of T cells and NK cells and then altering the number or activity of immune suppressor cells, including MDSCs, Tregs, and HLA-DR cells (83). Exosomes are also involved in different soluble factors such as check-point receptor ligands (PD-L1), inhibitory cytokines (IL-10 and TGF-β1), death receptor ligands (FasL), ectoenzymes, and prostaglandin E2 responsible for antitumor immunity in the tumor microenvironment (84, 85). In lymphocytes and NK cells, exosomes are associated with disease stage and activity in patients with HNC, suggesting that plasma exosomes may be related to HNC progression (86). In cancer, exosome signaling may affect the immune system by inhibiting the maturity of antigen-presenting cells and TDEs that carry and transfer tumor antigens to antigen-presenting cells, thereby inducing T cell- or NK cell-dependent immune responses (87). In a similar study, TDEs containing FasL and tumor necrosis factor α were found to induce T-cell apoptosis, thereby establishing an immunosuppressive tumor microenvironment to support tumor progression (88). TDEs derived from multiple HNCC cell lines induce a suppressive phenotype in CD8^+^ T cells through galectin-1, indicating that tumor-derived immunosuppressive exosomes may be potential therapeutic targets for preventing T-cell dysfunction and enhancing antitumor immune responses (89). As previously mentioned, the levels of oral SCC-derived FasL^+^ microvesicles are correlated with tumor burden, and the FasL^+^ microvesicles are involved in mediating apoptosis in activated T lymphocytes via receptor and mitochondrial pathways (51). A recent study showed that CD4^+^CD39^+^ Tregs produce adenosine by exposure to CD39^+^CD73^+^ exosomes from plasma in patients with HNSCC, thereby supporting tumor immune escape (90). Additionally, a recent study showed that oxygen pressure regulates the tumor microenvironment by altering exosomal miRNAs, which subsequently regulate the *miR-21*/phosphatase and tensin homolog (PTEN)/PD-L1 axis (91).

### HPV-Positive and -Negative Exosomes

The molecular and functional profiles of exosomes from HPV-positive and -negative HNSCC are different. An early study demonstrated that HNSCC-derived exosomes have different effects on the immune system in HPV-positive and -negative HNSCC. However, there are no differences in suppressive CD4^+^ and CD8^+^ T cells between HPV-positive and -negative exosomes, although the responses of human monocyte-derived dendritic cells (DCs) and mature DCs to exosomes are different. HPV-positive exosomes promote DC maturation, whereas HPV-negative exosomes suppress DC maturation. HPV-negative exosomes suppress the expression of antigen processing machinery, whereas HPV-positive exosomes do not (67). HPV-positive exosomes that promote DC maturation may also stimulate antitumor immune responses, thereby improving clinical outcomes in patients with HPV-positive HNSCC (67). HPV has been shown to utilize host exosomes for cell-cell communication and to induce EGFR expression and AKT signaling in recipient cells. One study found that exosomal NAP1 derived from oral cancer cells can promote the activation of NK cells by increasing the expression and phosphorylation of IRF-3 (92).

### Fibroblasts

**Figure 3:**
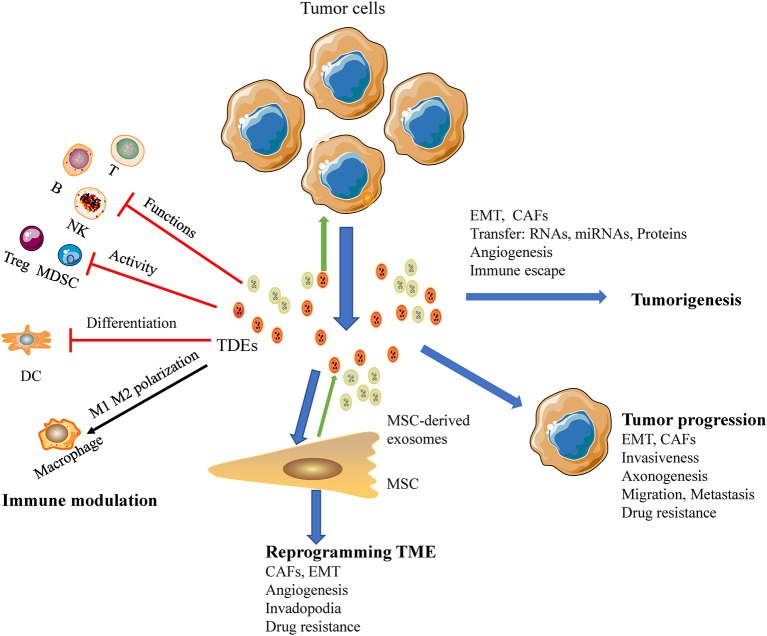
A macroscopic view of the functions of HNSCC-derived exosomes in the tumor microenvironment. Exosomes reduce tumor immunosurveillance by interfering with the immune system: (a) inhibit the functions of immune cells; (b) suppress the activity of Tregs, MDSCs; (c) interference the differentiation of DC; (d) TAMs polarization. HNSCC-derived exosomes mediated metastasis via stimulating proliferation of endothelial cells, EMT, CAFs, and promote angiogenesis as well as re-programming of the stromal compartment. Also HNSCC-derived exosomes influence cancer progression, promoting cancer cell growth, and invasive behavior of cancer cells and development of therapy resistance.

CAFs are particularly important for regulating tumor progression. Mesenchymal stem cells reprogrammed by TDEs mediate pro-angiogenic activity and convert stromal cells into CAFs; these cells are a major component of the tumor microenvironment and play key roles in promoting tumor progression (93). HNC-derived CAFs are innately resistant to cisplatin. Indeed, a study by Zhang showed that exosomal *miR-196a* derived from HNC causes cisplatin resistance by targeting CDKN1B and ING5, highlighting the roles of CAF-derived exosomal *miR-196a* in promoting cell proliferation and inhibiting cell apoptosis in the HNC microenvironment (60). Moreover, high expression of microfibril associated protein 5 in CAF-derived exosomes may contribute to the proliferation and metastasis of oral SCC via activation of the MAPK and AKT signaling pathways (94). Additionally, TDEs have been shown to deliver caveolin-1 to the tumor microenvironment to mediate the EMT and CAFs in tongue SCC (95). In a similar study, CAF-derived exosomal *miR-34a-5p* was found to be associated with oral cancer cell proliferation and metastasis in oral SCC (17). Thus, TDEs are emerging as potent mediators of the tumor microenvironment in HNSCC (Figure 3).

## Roles of TDEs in Regulating Cancer Progression and Metastasis

Many studies have evaluated the roles of TDEs in cancer initiation and progression. Numerous molecules in TDEs have been implicated in the initiation and progression of cancer cells or the tumor microenvironment (96). Indeed, TDEs can transform epithelial cells into cancerous cells, thereby initiating tumorigenesis. Studies have shown that TDEs reprogram the functions of recipient cells and facilitate premetastatic niche transformation to promote metastasis through cell-cell communication or autocrine signals. Additionally, TDEs facilitate tumor progression by delivery of factors necessary for sustaining tumor growth via utilizing autocrine or juxtacrine signaling (97). Furthermore, many studies have shown that RNA from TDEs can promote metastasis (57). The shuttling of miRNA molecules may cause tumorigenesis, tumor progression, and mRNA entry into recipient cells, resulting in protein translation and metastasis (44). The EMT has been implicated in cancer cell progression and metastasis, and TDEs deliver functional complexes by membrane fusion with recipient cells and binding of the recipient cell membrane receptors to promote the EMT (98).

Exosomes derived from hypoxic oral SCC cells promote cell migration and invasion by delivering *miR-21* to normoxic cells in HNSCC (15). Nakashima conducted a study indicating that *miR-200c-3p* had invasive capacity in the human oral SCC microenvironment (99). Moreover, CAFs contribute to the proliferation and metastasis of oral cancer cells via exosome-mediated paracrine *miR-34a-5p* signaling (17). Several studies have reported the heterogeneity of transforming growth factor (TGF) β signaling in oral cavity SCC. TGFβ expressed on the surface of TDEs can differentiate fibroblasts into myofibroblasts, thereby promoting tumor progression and metastasis in oral cavity SCC (100). Additionally, exosomes target different organs via variations in integrin molecules expressed on the surface. Released exosomes can be delivered to distant organs to promote oncogenic activity by constructing a suitable premetastatic tumor microenvironment for tumor migration. Thus, TDEs contribute to the development of this premetastatic niche and induce metastatic potential in recipient cells. A recent study showed that exosomes can transport EGFR to the liver to remodel the liver microenvironment (101). TDEs can transfer oncogenic EGFR to endothelial cells, triggering upregulation of vascular endothelial growth factor (VEGF) and autocrine VEGF in endothelial cells exposed to cancer cell-related MVs (64). Moreover, oral cancer cell-derived MVs promote endothelial cell angiogenesis through the Shh/RhoA signaling pathway (102). Additional experiments have demonstrated that exosomal *miR-150* promotes tumorigenesis by upregulating VEGF, and another study showed that HNSCC-derived exosomes stimulate angiogenesis *in vitro* and *in vivo* through functional reprogramming of endothelial cells (103). These studies suggest that exosomal miRNA could affect the biology of endothelial cells; then the exosomal miRNA induces angiogenesis in HNC through different regulation pathways.

TDEs modulate the immune response in tumor microenvironment interactions implicated in cancer progression. Oral SCC-derived exosomes induced M1-like TAMs polarized and promoted tumor metastasis (18). And tumor released exosomes containing EphrinB1 potentiate induce axonogenesis will promote tumor innervation in HNSCC (19). And the delivery of miRNA-21-abundant exosomes promote EMT-mediated M2-like polarization of TAMs may promote tumor progression of HNSCC (104). Another study demonstrated that exosomes derived from irradiated HNSCC cells can modify cancer cell movement and promote migration of recipient cells through AKT-signaling (105). In an additional series of experiments, exosomes were recruited to the plasma membrane of invadopodia, and knockdown of Rab27a decreased exosome secretion and extracellular matrix digestion associated with maturing invadopodia (35). The mechanisms through which TDEs regulate cancer progression and metastasis are illustrated in Figure 3.

## Therapeutic Application

Exosomes are cell-derived nanoparticles that have unique properties, such as low immunogenicity, strong ability to cross physiological barriers, good biodistribution and bioavailability, and reduced immunogenicity. Thus, TDEs may have important roles as potential vehicles for anticancer drugs. In addition, exosomes can be remodeled through their parental cells or supplemented with desired biological activity (106). Exosomes have been designed as promising therapeutic agents in the treatment of various cancers (107–109). Because patients with metastatic HNSCC have a poor prognosis and do not typically respond well to traditional therapies, efficient targeted delivery of conventional chemotherapeutic drugs may be facilitated by innovative approaches to engineering drug delivery systems, such as exosomes.

Drug carrier exosomes have been isolated from various types of cells, including HEK-293 cells, immature DCs, macrophages, and cancer cells. Different therapeutic agents, including proteins, small interfering RNAs (siRNAs), miRNAs, and targeted drugs, can be incorporated into exosomes via electroporation, chemical-based transfection, modification of parental cells, or direct incubation, thereby increasing bioactivity and achieving targeted delivery in patients. The James Graham Brown Cancer Center initiated a phase I clinical trial to test the therapeutic effects of plant exosomes in HNC (NCT01668849). In various types of cancer, exosomes have been shown to shuttle miRNAs and soluble proteins as therapeutic molecules into recipient cells and tissues (107, 110). A hypoxic microenvironment promotes the generation of *miR-21*-rich exosomes by oral SCC in a hypoxia-inducible factor (HIF)-1a- and HIF-2a-dependent manner, and these *miR-21*-rich exosomes then mediate migration and invasion behaviors. Restoration of *miR-21* expression in HIF-1a- and HIF-2a-depleted exosomes rescues oral SCC migration and invasion (15). Moreover, hypoxic TDEs mediate MDSC function through the *miR-21*/PTEN/PD-L1 axis in oral SCC (91). These findings indicate the therapeutic value of exosome inhibition for oral SCC treatment.

Anti-EGFR nanobodies anchored on extracellular vesicles via glycosyl phosphatidylinositol may improve the targeting ability of extracellular vesicles, highlighting the potential applications of these extracellular vesicles as a drug delivery system and new tool in EGFR-expressing tumor cells (111). The blood-brain barrier restricts drugs from entering into the brain, which can reduce the therapeutic effects of brain cancer treatments. Brain endothelial cell-derived exosomes can deliver anticancer drugs across the blood-brain barrier for the treatment of brain cancer. Additionally, exosome-based drug carriers can mediate permeability across the blood-brain barrier, enabling the drug to target cancer cells (112).

The main rational approaches for inhibiting exosome-mediated tumor-promoting potential have focused on blocking exosome release and suppressing the communication of tumor cells with recipient cells. TDEs are known to be abundantly secreted from cancer cells, making them potential targets for anticancer therapy. Direct targeting of the exosome release process has also been studied for the treatment of cancer. Exosome-delivered transient receptor potential polycystic 2 (TRPP2) siRNA markedly suppresses TRPP2 expression and inhibits the EMT, suggesting that exosome-TRPP2 siRNA may be an effective RNA-based gene therapy in the treatment of HNC (113). Invadopodia also enhance exosome secretion; accordingly, silencing the expression of invadopodia may suppress TDE biogenesis and release (114). Another potential strategy for exosomal dysregulation is the inhibition of exosome uptake. Treatment with heparin blocks the uptake of exosomes by oral SCC cells, thereby attenuating exosome-induced cancer progression to inhibit the growth and progression of oral SCC cells (20). However, the use of these reagents for eliminating exosomes is currently limited to research use only because these reagents may also induce off-target effects. Further studies are needed to explore the translational implications of exosome-targeted reagents.

Owing to the potential presence of TDEs and the unique biomarkers associated with these vesicles, TDEs may also have applications as vaccine immunotherapies (115). Antigen-presenting exosomes from B lymphocytes and DCs containing MHCI/II complexes could stimulate CD4^+^ and CD8^+^ T cells as therapeutic HPV vaccines (116). Additionally, HPV oncogenes play vital roles in HPV-induced carcinogenesis, and silencing of endogenous HPV E6/E7 expression affects both the contents and levels of MVs released from HPV-positive cancer cells (117). These findings indicate that inhibition of endogenous HPV E6/E7 expression may have therapeutic applications. HPV vaccines based on endogenously engineered exosomes for HNC have been evaluated in several phase I clinical trials (118). However, additional studies are required to determine the feasibility and safety of TDEs as cancer vaccines. One of the major challenges in developing this approach is establishing scalable, reproducible methods for exosome production. MSCs may have uses in exosome production at a clinically applicable scale owing to their ability of produce large amounts of exosomes.

Collectively, these studies suggest that exosome-based strategies may have many benefits over conventional drug regimens; however, there are some limitations and challenges to the use of exosomes. First, because TDEs contain genetic components from cancer cells, cancer cells may not be suitable parental cells for exosome targeting in clinical applications. Additionally, efficient loading of exosomes without significant alterations to the structure and content of exosomal membranes may be difficult. Overall, these reports indicate that exosomes may function as exceptional gene delivery vectors that are safe, efficient, organ-/cell-specific, and nonimmunogenic. Nevertheless, significant efforts are required to before clinical applications are feasible.

## Anti-Cancer Therapy Resistance

Current therapy options for HNSCC include surgery, radiotherapy, chemotherapy, anti-EGFR-antibody treatment, and immunotherapy (119). However, drug resistance remains a major obstacle for achieving successful curative treatment of cancer. Drug resistance includes endogenous drug resistance, innate drug resistance, and acquired drug resistance. Acquired drug resistance is a process through which cancer cells exposed to chemotherapy, radiation, or targeted therapy show reprogramming of their genome to acquire resistance to the therapy. Cancer cells may mitigate the effects of radiation and chemotherapy through different mechanisms (120, 121).

Given the important roles of exosomes in cellular communication and the tumor microenvironment, many studies have indicated that exosomes are involved in anticancer therapy resistance. In one mechanism, exosomes sequester cytotoxic drugs in intracellular vesicles and subsequently negate the effects of drugs within the cells (122). Owing to the nature of exosomes as mediators of cell-cell communication in the tumor microenvironment, these exosomes play important roles in therapy resistance by transferring various contents, such as miRNAs, mRNAs, DNAs, and proteins, to induce extrinsic therapy resistance (123). Similarly, exosomes also promote therapy resistance by transferring mRNAs, miRNAs, and other components. In addition, TDEs mediate therapy resistance by different mechanism, including improved DNA repair, anti-apoptotic signaling, or delivery of transporters to treatment-sensitive cells. As mediators of mesenchymal stem cells and the EMT, exosomes also promote tumor microenvironment-associated treatment resistance (123).

Exosomes derived from drug-resistant cancer cells mediate drug resistance through direct shuttling of drugs out of the cells (124). The AKT pathway is a frequently mutated oncogenic pathway in HNSCC and functions as a key regulator of radiation resistance and a major driver of cellular movement and migration (125). Exosomes derived from irradiated HNSCC cells trigger the AKT pathway to promote migration and increase chemotaxis in recipient cancer cells (105). Radiation therapy may increase the invasive and metastatic properties of HNSCC via release of an abundance of exosomes in hypoxic cancer tissue after radiotherapy (126–128). In patients with melanoma receiving PD-1 blockade therapy, the level of circulating exosomal PD-L1 correlates with tumor burden and response to therapy (129). The PD-1/PD-L1 immune checkpoint signaling axis exhibits remarkable responses in platinum-refractory recurrent or metastatic HNSCC; PD-L1-containing TDEs, which transfer functional PD-L1 and inhibit immune responses, may be regulators and biomarkers of resistance to PD-1 blockade therapy. In addition, exosomes derived from cisplatin-resistant HNSCC cells deliver *miR-21* to parental cells and induce cisplatin resistance, suggesting that these exosomes may function primarily through gene regulation (130). TDEs have been implicated in contributing to drug resistance in HNSCC (Figure 3).

## Future Implications

TDEs contain numerous bioactive cellular molecules and genetic characteristics, enabling them to alter the functions of recipient cells and the tumor microenvironment. Accordingly, TDEs play important regulatory roles in cancer. Indeed, TDEs are involved in many aspects of intercellular substance transmission and signal transfer, contributing to the initiation, development, metastasis, treatment resistance, and immunosuppression of HNSCC. In HNSCC, TDEs may serve as potential clinical biomarkers of progression or responses to therapy owing to their various functional contents (proteins, genes) and elements of the parental cancer cells. Nevertheless, exosomes have not yet been applied in the treatment of HNSCC. Further studies are needed to elucidate the molecular mechanisms involved in the release of exosomes and to explore the clinical applications of these vesicles. Understanding how cancer cells utilize TDEs to promote cancer growth and progression may lead to the development of novel therapies for HNSCC. Therefore, much work is needed to establish exosome-based therapies for the treatment of HNSCC.

## Author Contributions

CX, FS, and NX: review concept, review design, interpretation, manuscript preparation, and manuscript review. YZ, JL, and QW: manuscript preparation and manuscript review.

### Conflict of Interest Statement

The authors declare that the research was conducted in the absence of any commercial or financial relationships that could be construed as a potential conflict of interest.
